# Consumption of Vitamin K and Vitamin A Are Associated With Reduced Risk of Developing Emphysema: NHANES 2007–2016

**DOI:** 10.3389/fnut.2020.00047

**Published:** 2020-04-21

**Authors:** Tianjiao Shen, Milan Bimali, Mohammed Faramawi, Mohammed S. Orloff

**Affiliations:** ^1^Department of Epidemiology, University of Arkansas for Medical Sciences, Little Rock, AR, United States; ^2^Department of Biostatistics, University of Arkansas for Medical Sciences, Little Rock, AR, United States; ^3^Winthrop P. Rockefeller Cancer Institute, University of Arkansas for Medical Sciences, Little Rock, AR, United States

**Keywords:** COPD, emphysema, lung disease, green vegetable, NHANES data

## Abstract

Chronic obstructive pulmonary disease (COPD) comprising of emphysema and chronic bronchitis are the most common chronic respiratory diseases that impart a huge economic and clinical burden. Factors other than smoking and air pollutants can cause inflammation and emphysematous changes in the lung airspaces or alveoli have been understudied. Using a cross-sectional study design, we assessed the association of dark green vegetables, vitamin K and Vitamin A with emphysema status among adults at U.S. These nutrients have a role in lung biology. A complete case NHANES data (*n* = 17,681) was used. After adjusting for modifiable and non-modifiable confounders, consumption of recommended amounts of vitamin K was associated with 39% decrease in odds (Odds Ratio: 0.61; 95% CI: 0.40–0.92, *P*-val: 0.02) of emphysema. Similarly consumption of recommended amounts vitamin A dose was associated with 33% decrease in odds (Odds Ratio: 0.67; 95% CI: 0.44–1.00, *P*-val: 0.05) of emphysema. Vitamin K shows an inverse association suggesting that it may be important in slowing the emphysematous process. Vitamin A is important in maintaining the anti-inflammatory process. Together vitamin K and vitamin A are important in the lung health.

## Introduction

Chronic obstructive pulmonary disease (COPD) and asthma are the most common chronic respiratory diseases that imparts a huge economic and clinical burden in the health sectors (1, 2). In the US, COPD costs $30 billion in direct health care expenditures and $20 billion in indirect expenditure (1). COPD is the first leading cause of disability and 3rd leading cause of death in the U.S (3, 4). COPD comprises of two heterogeneous subtypes: emphysema and chronic bronchitis (5). Emphysema is associated with structural changes, including abnormal and permanent enlargement or damage of the airspaces distal to the terminal bronchioles, while chronic bronchitis results from inflammation of the lining of the bronchial tubes (6). Reported risk factors associated with the emphysematous changes such as gender, tobacco smoke, obesity, and exposure to air pollutants (7, 8) do not completely explain development of emphysema.

Even though smoking is a key risk factor for the development and progression of the COPD, many patients with this obstructive lung function are never-smokers. Along these lines, recent studies have shown that lack or excess of specific nutrition may also play a role in developing lung diseases. Some of these studies provide evidence that vegetable intake may have potential benefits in the biology of the lung diseases (9–26). However, data is still limited with regards to specific constituents or micronutrients in vegetables that are either risk factors for or have beneficial roles on the development of the COPD phenotypes and more specifically emphysema. Trace elements have also been linked to harmful effects on lung function (27). For example cadmium in tobacco smoke may contribute to the development of pulmonary emphysema. However, there is poor understanding of the mechanisms behind the pathogenic role of cadmium in lung diseases.

Studies have shown that inflammation plays a role in the exacerbation of COPD (28), and chronic inflammation can cause damage to the lung airways and tissues (29), resulting to irreversible emphysematous changes in the lung alveoli (30, 31). Reports have shown that micronutrients such as vitamins E and C play a role in strengthening the immune system (32, 33). A meta-analysis has shown that high vitamin C intake levels have a significant protective effect against lung diseases e.g., lung cancer (34, 35). Although vegetables have been shown to have beneficial nutrients that can aid healthy lung structure (34, 36, 37), more research is warranted to link specific nutrients in the greens that can help protect the alveoli from developing emphysematous lung injuries. Since vitamin K is mostly obtained from dark green vegetable, it is highly likely that it may have an impact on the biology of lung airway or alveoli structure (38, 39). Low intake of vitamin A also from dark green vegetables has been associated with COPD in different populations (40–42) and a few studies have shown the potential role of vitamin A in maintaining the integrity of the lung epithelium, non-emphysematous state and exerts anti-inflammatory effects (43, 44). Therefore, we hypothesize that dark green vegetables including vitamin K and vitamin A consumptions may be associated with emphysema.

Thus, the objective of this study is to (separately) examine the association between dark green vegetables, and risk of emphysema; and the association between both vitamins K and A with the risk of developing emphysema.

## Methods

### Study Design and Population

A cross-sectional design was used in this study. Data was acquired from the National Health and Nutrition Examination Survey (NHANES), which is a program designed to assess the health and nutritional status of adults and children in the United States (45). Data for the current analysis are from the 2007–2016 survey cycles. The sample for the survey was selected to represent the U.S. population of all ages which included adults aged ≥20 in U.S who completed the questionnaire. The questionnaire collected information on demographics (gender, age, race, education, household income, and marital status), behavioral factors (smoking status and heavy drinking), BMI, medical conditions, and 24 h food recall. About 12,000 persons every 2-year cycle were asked to participate, and the average response rate was 87.5% (45). Of the 12,000 NHANES respondents, subsamples were randomly selected to participate in a variety of survey components (e.g., dietary survey, laboratory examination) according to the NHANES protocol. From 2007 to 2012, one-half of the total respondents were sampled to complete the survey components of interest in our study; from 2013 to 2016, one-third of total respondents were sampled.

### Measures

#### Outcome

Emphysema was assessed based on self-reported Yes/No response to the questions: Has a doctor or other health professional ever told you that you have emphysema?

### Exposure

#### Dark-Green Vegetables and Vitamins

A 24-h dietary/food recall was used to collect dietary intake data. Food intake categories are defined by food code following the USDA Food and Nutrient Database for Dietary Studies (46). Based on previously reported links between vitamin K, vitamin A as well as dark green vegetable on lung health makes them suitable exposures for assessing development of emphysema. Dark-green vegetable is one of eight subtypes of vegetable as shown in Table 1. The estimated weight of the dark-green vegetable intake (in grams) was obtained from the 24 h dietary recall. Dark green vegetables is the main source of vitamin K and vitamin A can also be found in the dark green vegetables. Dichotomous (45) measures of vitamins and dark-green vegetable intake, were obtained by categorizing the dietary intake of vitamins into Recommended Dietary Allowances (RDAs) were not consumed (No) and RDAs were consumed (Yes) and “weight of the dark-green vegetables intake” into 0 gram (Intake: No) and >0 gm (Intake: Yes). RDAs for vitamins were stratified by age, gender, pregnancy, and lactation according to National Institutes of Health's website (https://ods.od.nih.gov).

**Table 1:** Subtypes of vegetables.

**Vegetable**
(1) White potatoes and Puerto Rican starchy vegetables,
(2) Dark-green vegetables,
(3) Deep-yellow vegetables,
(4) Tomatoes and tomato mixtures,
(5) Other vegetables,
(6) Vegetables and mixtures mostly vegetables baby food,
(7) Vegetables with meat, poultry, fish, and
(8) Mixtures mostly vegetables without meat, poultry, fish.

#### Covariates

Age, gender, race/ethnicity, BMI, education level, marital status, smoking status, alcohol (heavy drinking status) intake, cadmium exposure, energy intake, and physical activity were identified as potential confounders.

The demographic data (age, race/ethnicity, education, marital status) was collected by employing a questionnaire. Age was reported in years. Gender was reported as either “Male” or “Female.” Race was grouped into five categories: Non-Hispanic White, Non-Hispanic Black, Mexican American, other Hispanic, and other races.

Body Mass Index (kg/m^2^) was calculated based on height and weight. Education was grouped into 4 categories: less than high school, high school graduate/GED or equivalent, some college or AA degree, and college graduate or above. Marital Status was grouped into two categories: single (widowed/divorced/separated/never married) and married (married or living with partner). Smoking status consisted of four categories: non-smoker, former smoker, someday smoker, and every day smoker, based on two questions “Did you smoke at least 100 cigarettes in life? (SMQ020)” and “Do you now smoke cigarettes? (SMQ040).” Non-smokers were defined as individuals who smoked less 100 cigarettes in life; Former-Smokers were those who smoked at least 100 cigarettes in their life and did not smoke currently; Someday smokers were adults who had smoked at least 100 cigarettes in their lifetime, who smoked now, but did not smoke every day; Every day smokers were adults who had smoked at least 100 cigarettes in their lifetime, and who now smoked every day. Heavy drinking was defined as “Yes” response to “Ever have 5 or more drinks every day?” for both male and female from 2007 to 2010 and to “Ever have 4/5 or more drinks every day?” for female and male separately from 2011 to 2016. The number of heavy drinking day and Average number alcoholic drinks/day in the past 12 months were calculated by questionnaire to provide detailed information on alcohol use behavior. Cadmium exposure (μg/L) was obtained from analyzing blood samples. Energy intake (kilocalorie) was defined as total energy of foods consumed in the 24-h dietary recall.

### Statistical Analysis

After excluding participants with missing observations there were 17681 observations (2007–2008: 4,628, 2009–2010: 4,780, 2011–2012: 4,014, 2013–2014: 2,186, and 2015–2016: 2,077) based on NHANES data.

Descriptive measures were calculated and reported as mean (standard error) for continuous variables, and as frequency (or percentage), weighted frequency for categorical variables and lung emphysema status. Bivariate association between categorical variables were assessed using Rao-Scott's modified Chi-square test. Continuous variables were compared using linear regression adjusted by interview weights. The association between vitamins and odds of emphysema were estimated separately using logistic regression. To account for the sampling design, the models were adjusted for the sampling weight, sampling cluster, and sampling strata. Multivariate modeling was done by regressing response (emphysema) on vitamins intake, dark green vegetable together with non-modifiable confounders—age, gender, ethnicity; and modifiable confounders—BMI, education level, marital status, smoking status, alcohol (heavy drinking status) intake, cadmium exposure, and total energy intake. Ninety-five percentage confidence interval was reported together with the odds ratio estimates. The analysis was done in SAS 9.4 (SAS Institute).

## Results

Table 2 summarizes the characteristics of the study population. The complete case analysis involved 17,681 respondents. There were 396 (1.82%) participants with self-reported emphysema.

**Table 2:** Characteristics of Study Population, National Health and Nutrition Examination.

**Variable**	**Levels**	**Overall** **(*n* = 17,681)**	**Emphysema**	
			**Yes (*n* = 391)**	**No (*n* = 17,290)**
Vitamin K	Yes	4,976 (32.06) 61,371,378	75 (18.79) 652,992	4,901 (32.30) 60,718,385
Vitamin A	Yes	4,232 (26.78) 51,264,497	75 (7.19) 624,915	4,157 (12.59) 50,639,582
Dark-green Veg Intake	Yes	1,942 (12.49) 23,916,374	27 (7.19) 250,078	1,915 (12.59) 23,666,296
Gender	Female (1)	8,768 (48.43) 92,724,512	244 (58.11) 2,019,916	8,524 (48.26) 90,704,595
	Male (2)	8,913 (51.56) 98,707,458	147 (41.89) 1,456,217	8,766 (51.74) 97,251,242
Race/Ethnicity	Non-hispanic white (3)	8,097 (69.38) 132,824,555	286 (84.98) 2,954,148	7,811 (69.10) 129,870,407
	Non-hispanic black (4)	3,579 (10.55) 20,197,763	47 191,819 (5.52)	3,532 20,005,943 (10.64)
	Mexican American (1)	2,679 (8.13) 15,569,372	14 (1.72) 59,681	2665 (8.25) 15509691
	Other hispanic (2)	1,787 (5.30) 10,150,921	24 86,160 17,435 (2.48)	1,763 (5.35) 1,006,4761
	Other race - including multi-racial (5)	1,539 (6.63) 12,689,360	20 (5.30) 184,325	1,519 (6.65) 12,505,035
Heavy drinking status	No (0)	1,5019 (85.53) 163,724,962	246 (64.23) 2,232,619	14,773 (85.92) 161,492,343
	Yes (1)	2,662 (14.47) 27,707,008	145 (35.77) 1,243,514	2,517 (14.08) 26,463,494
Smoking status	Never smoked (0)	9,668 (55.21) 105,683,001	27 (8.64) 300,201	9,641 (56.07) 105,382,800
	Former smoker (1)	4,369 (25.22) 48,274,092	189 (50.06) 1,740,234	4,180 (24.76) 46,533,859
	Someday smoker (2)	654 (3.69) 7,065,257	10 (3.44) 119,527	644 (3.70) 6,945,729
	Every day smoker (3)	2,990 (15.89) 30,409,620	165 (37.86) 1,316,171	2,825 (15.48) 29,093,449
Education level	Less than high school	4,347 (15.29) 29,262,368	168 (34.19) 1,188,496	4,179 (14.94) 28,073,871
	High school/GED equivalent	4,047 (22.23) 42,559,180	98 (26.08) 906,455	3,949 (22.16) 41,652,725
	Some college/AA equivalent	5,161 (32.10) 61,442,603	86 (26.11) 907,731	5,075 (32.21) 60,534,872
	College graduate or above	4,126 (30.39) 58,167,819	39 (13.62) 473,450	4,087 (30.70) 57,694,369
Marital status	Married/living with partner	10,578 (63.09) 120,766,233	190 (53.22) 1,849,998	10,388 (63.27) 118,916,235
	Single	7,103 (36.91) 70,665,737	201 (46.78) 1,626,135	6,902 (36.73) 69,039,602
Age	Mean (std. error)	47.40 (0.29)	47.12 (0.29)	62.89 (0.75)
BMI		29.08 (0.10)	29.07 (0.10)	29.88 (0.55)
Cadmium		0.47 (0.01)	0.46 (0.01)	0.92 (0.03)
Total energy intake		2,161.82 (9.80)	2,164.89 (10.01)	1,995.73 (59.43)

*The unit of BMI, Cadmium, and Total energy intake are (kg/m2), (ug/L), and kilocalorie, respectively. For marital status single includes: Widowed/Divorced/ Separated/Never Married.The values for categorical variables are raw frequency, (percentage), and weighted frequency, respectively. Apart from Dark Green Vegetable Intake and BMI, all the other variables had P-value < 0.05 based on bivariate association for categorical variables and linear regression for continuous variables*.

Overall, 32.06% participants recommended vitamin k consumption; 26.78% participants recommended vitamin A consumption; 12.49 % of respondents reported dark green vegetable consumption. The recommended vitamin K consumption; recommended vitamin A consumption; and dark green vegetable consumption among those with self-reported emphysema was 18.79, 7.19, and 7.19%, respectively.

Overall, 48.43% were females; Non-Hispanic Whites (69.38%), and Non-Hispanic Blacks (10.55%) were two most commonly reported race/ethnicity categories; 63.09% were married or living with partner; some college or advanced associate equivalent (32.10%) and college degree or above (30.39%) and were two most commonly reported education level; 14.47% were heavy drinkers; 55.21% never smoked, and 25.22% were former smokers (two most frequent smoking status). The mean age, BMI, blood cadmium level, and total energy intake were 47.40 years, 29.08 (Kg/m^2^), 0.47 (μg/L), and 2,161.82 (kilocalorie), respectively.

Among those with self-reported emphysema, 58.11% were females; Non-Hispanic Whites (84.98%), and Non-Hispanic Blacks (5.52%) were two most commonly reported race/ethnicity categories; 53.22% were married or living with partner; some college or advanced associate equivalent (26.11%) and college degree or above (13.62%) were two most commonly reported education level; 35.77% were heavy drinkers; 50.06% were former smokers, and 37.86% were everyday smokers (two most frequent smoking status). The mean age, BMI, blood cadmium level, and total energy intake were 47.12 years, 29.07 (Kg/m^2^), 0.46 (μg/L), and 2,164.89 (kilocalorie), respectively.

Among those with self-reported emphysema, 58.11% were females; Non-Hispanic Whites (84.98%), and Non-Hispanic Blacks (5.52%) were two most commonly reported race/ethnicity categories; 53.22% were married or living with partner; less than high school (34.19%), high school or GED equivalent (26.08%), and some college or AA equivalent (26.11%) were three most commonly reported education level; 35.77% were heavy drinkers; 50.06% were former smokers and 41.09% were never smokers (two most frequent smoking status). The mean age, BMI, and blood cadmium level was 62.41 years, 29.72 (Kg/m^2^), and 0.95 (μg/L), respectively.

### Relationship Among Dark-green Vegetables Intake, Vitamins, and Emphysema

The summary of findings examining the association between dark-green vegetables intake, vitamins, and risk of emphysema are summarized in Tables 3, 4. After adjusting for modifiable and non-modifiable confounders, consumption of recommended vitamin K was associated with 39% decrease in odds (Odds Ratio: 0.61; 95% CI: 0.40–0.92, *P*-val: 0.02) of emphysema compared to those who consumed less than the recommended vitamin K dose. The association between consumption of recommended vitamin k and emphysema changed marginally when consumption of dark green vegetable was incorporated in the model (Odds Ratio: 0.62; 95% CI: 0.44–0.87, *P*-val: 0.01). Dark-green vegetable consumption by itself, after adjusting for modifiable and non-modifiable confounders, was associated with 30% decrease in odds of emphysema, the findings did not attain statistical significance (Odds Ratio: 0.70; 95% CI: 0.32–1.52, *P*-val: 0.36).

**Table 3:** Relationship among dark-green vegetables intake, vitamin K, and emphysema.

	**Vitamin K**	**Dark green veg**	**Note**
Model 1	0.62 (0.44,0.87) *P*-val: 0.01	0.96 (0.45,2.06) *P*-val: 0.92	Vitamin K and dark green veg are significantly associated (*P*-val: < 0.0001)
Model 2	–	0.70 (0.32,1.52) *P*-val: 0.36	
Model 3	0.61 (0.40,0.92) *P*-val: 0.02	–	

*The estimates are the odds of lung disease associated with consumption of recommended vitamins dose compared to those that did not consume recommended vitamin dose*.

*The model is adjusted for: Adjusted for Age, gender (reference = “Male”), and ethnicity (reference = “Non-Hispanic White”), BMI, smoking status (reference = “Never Smoked”), heavy drinking status (reference = “No”), education level (reference = “College Graduate or Above”), marital status (reference = “Married or Living with Partner”), blood cadmium level, and total energy intake*.

**Table 4:** Relationship among dark-green vegetables intake, vitamin A, and emphysema.

**Vitamin**	**Vitamin A**	**Dark green veg**	**Note**
Model 1	0.69 (0.47,1.00) *P*-val: 0.05	0.75 (0.35,1.61) *P*-val: 0.46	Vitamin A and dark green veg are significantly associated (*P*-val: < 0.0001)
Model 2	–	0.70 (0.32,1.52) *P*-val: 0.36	
Model 3	0.67 (0.44,1.00) *P*-val: 0.05	–	

*The estimates are the odds of lung disease associated with consumption of recommended vitamins dose compared to those that did not consume recommended vitamin dose*.

*The model is adjusted for: Adjusted for Age, gender (reference = “Male”), and ethnicity (reference = “Non-Hispanic White”), BMI, smoking status (reference = “Never Smoked”), heavy drinking status (reference = “No”), education level (reference = “College Graduate or Above”), marital status (reference = “Married or Living with Partner”), Cadmium exposure, and total energy intake*.

Vitamin A was another vitamin strongly associated with dark-green vegetables. After adjusting for the effect of modifiable as well as non-modifiable confounders consumption of recommended vitamin A was associated with 33% decrease in odds (Odds Ratio: 0.67; 95% CI: 0.44–1.00, P-val: 0.05) of emphysema compared to those who consumed less than recommended vitamin A dose. The estimates were similar when consumption of dark green vegetable was incorporated in the previous model (Odds Ratio: 0.69; 95% CI: 0.47–1.00, *P*-val: 0.05).

## Discussion

While we observed an inverse association between vegetables intake and lung disease as previously reported, for the first time we show an inverse association between vitamin K intake and vitamin A intake with emphysema. A cross-sectional study design was used to assess the association of vitamin K, dark green vegetables and Vitamin A with reported emphysema status derived from NHANES. After adjusting for relevant confounders, consumption of recommended amounts of vitamin K was associated with reduced risk of developing emphysema. Similarly consumption of recommended amounts of vitamin A was associated with reduced risk of developing emphysema.

A previous study has shown that reduced vitamin K status in COPD patients was associated with high mortality rates (39). The study also showed that elastin degradation is accelerated in chronic COPD and is partially regulated by Matrix Gla Protein (MGP), via a vitamin K-dependent pathway. Therefore, vitamin K has a potential role in COPD pathogenesis. Interestingly, we were able to show that consumption of recommended amounts of vitamin K is inversely associated with emphysema status. Therefore, vitamin K may impart a protective role by reducing or slowing down the emphysematous damage.

Specific vitamins and certain trace elements like iron, zinc, copper and selenium work in synergy to support the protective activities of the immune cells. Depleted amounts of these micronutrients can impact antibody production and affect the inflammation process or lung health (32). Along these lines analysis of the NHANES data showed an inverse association between vitamin A and emphysema. Therefore, supplementation with appropriate amounts of vitamin A can exhibit an anti-inflammatory related responses important to support regulated lung airway function (47, 48).

A population-based prospective cohort of Swedish men showed that high consumption of fruits and vegetables was inversely associated with reduced COPD incidence in both current and ex-smokers but not in never-smokers (16). Interestingly, women from the same population-based prospective cohort who consumed fruits and vegetables long-term showed that fruits but not vegetables was inversely associated with COPD incidence independent of the smoking status. It was clear from our analysis of the NHANES dataset that vitamin K as well as vitamin A likely from the dark green vegetables were inversely association with emphysema.

The strengths of our study are relatively large sample size, high generalizability, and comprehensive questionnaire data. However, there are several limitations. The most important one is that 24-h dietary recall potentially results in misclassification of exposure because 24-h dietary recall may be unrepresentative to the long-term dietary behaviors. However, the analysis suggested this misclassification is non-differential. Of all emphysema patients, 75.40% reported the 24-h diet is similar to usual, 7.60% consumed food more than usual, and 16.99% consumed less than usual. The distribution of comparing 24-h diet to “usual” among controls was similar as emphysema cases. Among controls, 76.41% reported the 24-h diet is similar to “usual,” 7.55% more than “usual,” and 16.06% less than “usual.” Thus, this non-differential misclassification due to 24-h dietary recall could bias the result toward the null. The outcome was defined as Yes response to “Has a doctor or other health professional ever told you that you have emphysema?” Although under-diagnosis of emphysema could happen, to some degree respondents either with or without emphysema are unlikely to misreport their history of diagnosis. Thus, the misclassification of outcome is likely non-differential which biases the result toward the null. The association between dark-green vegetable intake and emphysema would be stronger if the above misclassification of exposure and outcome did not occur.

It is important to mention that with regards to monitoring lung function, there is no gold standard for measuring symptoms related to proper lung function, since none of the available methods is optimal in all regards (49). Some of the tests used in questionnaires hardly reflect the heterogeneity, variability, and severity of COPD or its phenotypes e.g., emphysema, as well as the numerous confounding factors contributing to the clinical presentation of the disease. This can lead to misclassification of outcome (49). Generally, COPD which encompasses chronic bronchitis and emphysema is commonly underdiagnosed. Along these lines, a previous study using NHANES dataset showed that 5.2% of US adults aged 40–79 reported being diagnosed with COPD during 2007–2012 (50). This study determined that multiple factors are associated with self-reported COPD diagnosis where the number of reported respiratory symptoms, probably non-related to COPD, had the strongest association. But after controlling for other factors, having mild lung obstruction was not associated with being diagnosed with COPD. Further, the overall COPD prevalence among US adults aged 40–79 years varied between 10.2 and 20.9% based on whether pre- or post-bronchodilator values were used and which diagnostic criterion was applied (51). The overall prevalence decreased by approximately 33% when airflow limitation was based on post-bronchodilator as compared to pre-bronchodilator spirometry, regardless of which diagnostic criterion was used (51).

A recent study showed that among those with spirometry-defined obstruction, 72.0% (SE, 1.9) in NHANES 2007–2012 were undiagnosed (52). Further, using multivariate models, undiagnosed obstructive disease was consistently associated in both surveys (NHANES 2007–2012 and NHANES III) with self-reported good/excellent health status, lower comorbidity burden, higher lung function, and being of racial/ethnic minority. On the other hand there was no association between undiagnosed disease or healthier profile and education level in either survey NHANES 2007–2012 or NHANES III (OR = 0.94, 95% = 0.61–1.44, where “high school and beyond” was compared to “below high school”) (52). Although educational level of self-reporting diagnosed emphysema patients were different from control group, we suggest that the bias due to under diagnosis is non-differential, weakening the association between dark-green vegetable intake and emphysema.

Also, information bias is likely because of retrospective data collection and the intrinsic limitations of the 24-h dietary/food recall that the participants were subjected to. Potentially, participants may over-report their intake of fruits and vegetables, hence a recall bias (53). Unfortunately, data is not available to assess whether differential misclassification of dark green vegetable intake occurred due to misreporting and to determine the direction and magnitude of misclassification bias.

Although we controlled for well-documented risk factors namely age, gender, and race, BMI, education level, marital status, smoking status, alcohol intake, and blood cadmium level; residual confounding may still persist in this study. Food except for vegetables and fruits may contain unknown biologically active compounds, which may be correlated with vegetables and fruits but were not controlled for in the study. In addition, there is usually a complicated relationship between foods and food subtypes that may potentially modify the direction of association with lung diseases. Important to note that the inferences stayed same after including physical activity in the model.

In conclusion, the results suggest that consumption of dark green vegetable and recommended amounts of micronutrient such as vitamin K and vitamin A can help slow or halt the emphysematous process. Clearly, dietary or protective interventions with specific micronutrients early in life may promote a healthy lung and also curb emphysematous injury. More prospective cohorts and well-designed clinical trials are needed to promote the transition of individualized nutrient interventions into health policy. A replication using an expanded sample size followed by functional analysis would be beneficial.

## Data Availability Statement

Publicly available datasets were analyzed in this study. This data can be found here: https://wwwn.cdc.gov/nchs/nhanes/Default.aspx.

## Author Contributions

All authors listed have made a substantial, direct and intellectual contribution to the work, and approved it for publication.

## Conflict of Interest

The authors declare that the research was conducted in the absence of any commercial or financial relationships that could be construed as a potential conflict of interest.

## References

[1] Toy EL Gallagher KF Stanley EL Swensen AR Duh MS. The economic impact of exacerbations of chronic obstructive pulmonary disease and exacerbation definition: a review. COPD. (2010) 7:214–228. 10.3109/15412555.2010.481697 20486821

[2] Nurmagambetov T Kuwahara R Garbe P. The economic burden of asthma in the United States, 2008–2013. Ann Am Thorac Soc. (2018) 15:348–56. 10.1513/AnnalsATS.201703-259OC 29323930

[3] Heron MP. Deaths: leading causes for 2013. Natl Vital Stat Rep. (2016) 65:1–95. 26906146

[4] Statistics NCfH Health, United States, 2015: With Special Feature on Racial and Ethnic Health Disparities (2016). 27308685

[5] Marsh SE Travers J Weatherall M Williams MV Aldington S Shirtcliffe PM. Proportional classifications of COPD phenotypes. Thorax. (2008) 63:761–7. 10.1136/thx.2007.089193 18728201 PMC2862964

[6] Thurlbeck WM Henderson JA Fraser RG Bates DV Chronic obstructive lung disease. A comparison between clinical, roentgenologic, functional and morphologic criteria in chronic bronchitis, emphysema, asthma and bronchiectasis. J Occup Environ Med. (1970) 12:533 10.1097/00005792-197003000-00001

[7] Gibson P Simpson J. The overlap syndrome of asthma and COPD: what are its features and how important is it? Thorax. (2009) 64:728–35. 10.1136/thx.2008.108027 19638566

[8] Beasley R Semprini A Mitchell EA. Risk factors for asthma: is prevention possible? Lancet. (2015) 386:1075–85. 10.1016/S0140-6736(15)00156-7 26382999

[9] Amararathna M Johnston M Rupasinghe H. Plant polyphenols as chemopreventive agents for lung cancer. Int J Mol Sci. (2016) 17:1352. 10.3390/ijms17081352 27548149 PMC5000748

[10] Dreher M. Whole fruits and fruit fiber emerging health effects. Nutrients. (2018) 10:1833. 10.3390/nu10121833 30487459 PMC6315720

[11] Garcia-Larsen V Arthur R Potts JF Howarth PH Ahlström M Haahtela T. Is fruit and vegetable intake associated with asthma or chronic rhino-sinusitis in European adults? Results from the global allergy and asthma network of excellence (GA2LEN) survey. Clin Transl Allergy. (2017) 7:3. 10.1186/s13601-016-0140-9 28149501 PMC5273849

[12] Grundy A Poirier AE Khandwala F McFadden A Friedenreich CM Brenner DR. Cancer incidence attributable to insufficient fruit and vegetable consumption in Alberta in 2012. CMAJ Open. (2016) 4:E760. 10.9778/cmajo.20160037 28018892 PMC5173484

[13] Guilleminault L Williams JE Scott AH Berthon SB Jensen M Wood GL. Diet and asthma: is it time to adapt our message? Nutrients. (2017) 9:1227. 10.3390/nu9111227 29117118 PMC5707699

[14] Hosseini B Berthon BS Wark P Wood LG. Effects of fruit and vegetable consumption on risk of asthma, wheezing and immune responses: a systematic review and meta-analysis. Nutrients. (2017) 9:341. 10.3390/nu9040341 28353635 PMC5409680

[15] Kaluza J Larsson SC Orsini N Linden A Wolk A. Fruit and vegetable consumption and risk of COPD: a prospective cohort study of men. Thorax. (2017) 72:500. 10.1136/thoraxjnl-2015-207851 28228486

[16] Kaluza J Wolk A Harris HR Linden A. Long-term consumption of fruits and vegetables and risk of chronic obstructive pulmonary disease: a prospective cohort study of women. Int J Epidemiol. (2018) 47:1897–909. 10.1093/ije/dyy178 30239739

[17] Kromhout D Spaaij CJK de Goede J Weggemans RM. The 2015 Dutch food-based dietary guidelines. Eur J Clin Nutr. (2016) 70:869. 10.1038/ejcn.2016.52 27049034 PMC5399142

[18] Meteran H Thomsen SF Miller MR Hjelmborg J Sigsgaard T Backer V. Self-reported intake of fruit and vegetables and risk of chronic obstructive pulmonary disease: a nation-wide twin study. Respir Med. (2018) 144:16–21. 10.1016/j.rmed.2018.09.013 30366579

[19] Du H Li L Bennett D Yang L Guo Y Key TJ. Fresh fruit consumption and all-cause and cause-specific mortality: findings from the China kadoorie biobank. Int J Epidemiol. (2017) 46:1444–55. 10.1093/ije/dyx042 28449053 PMC5837264

[20] Rauf A Imran M Butt MS Nadeem M Peters DG Mubarak MS. Resveratrol as an anti-cancer agent: a review. Crit Rev Food Sci Nutr. (2018) 58:1428–47. 10.1080/10408398.2016.1263597 28001084

[21] Seyedrezazadeh E Ansarin K Pour Moghaddam M Reza Vafa M Sharma S Kolahdooz F. Fruit and vegetable intake and risk of wheezing and asthma: a systematic review and meta-analysis. Nutr Rev. (2014) 72:411–28. 10.1111/nure.12121 24947126

[22] Stevens C Navarro-Rosenblatt D Chan DSM Abar L Vingeliene S Norat T. Fruits, vegetables and lung cancer risk: a systematic review and meta-analysis. Ann Oncol. (2015) 27:81–96. 10.1093/annonc/mdv381 26371287

[23] Tarrazo-Antelo AM Ruano-Ravina A Abal Arca J Barros-Dios JM. Fruit and vegetable consumption and lung cancer risk: a case-control study in Galicia, Spain. Nutr Cancer. (2014) 66:1030–7. 10.1080/01635581.2014.936951 25085257

[24] Voortman T Kiefte-de Jong JC Ikram MA Stricker BH van Rooij FJA Lahousse L. Adherence to the 2015 Dutch dietary guidelines and risk of non-communicable diseases and mortality in the rotterdam study. Eur J Epidemiol. (2017) 32:993–1005. 10.1007/s10654-017-0295-2 28825166 PMC5684301

[25] Wang M Qin S Zhang T Song X Zhang S. The effect of fruit and vegetable intake on the development of lung cancer: a meta-analysis of 32 publications and 20 414 cases. Eur J Clin Nutr. (2015) 69:1184–92. 10.1038/ejcn.2015.64 25920421

[26] Berthon B Wood L. Nutrition and respiratory health—feature review. Nutrients. (2015) 7:1618–43. 10.3390/nu7031618 25751820 PMC4377870

[27] Ganguly K Levanen B Palmberg L Akesson A Linden A. Cadmium in tobacco smokers: a neglected link to lung disease? Eur Respir Rev. (2018) 27:170122. 10.1183/16000617.0122-2017 29592863 PMC9488953

[28] George L Brightling CE. Eosinophilic airway inflammation: role in asthma and chronic obstructive pulmonary disease. Ther Adv Chronic Dis. (2016) 7:34–51. 10.1177/2040622315609251 26770668 PMC4707428

[29] Ito K Barnes PJ. COPD as a disease of accelerated lung aging. Chest. (2009) 135:173–80. 10.1378/chest.08-1419 19136405

[30] Ojo O Lagan AL Rajendran V Spanjer A Chen L Sohal SS. Pathological changes in the COPD lung mesenchyme–novel lessons learned from *in vitro* and *in vivo* studies. Pulm Pharmacol Ther. (2014) 29:121–8. 10.1016/j.pupt.2014.04.004 24747433

[31] Pirozzi CS Gu T Quibrera PM Carretta EE Han MK Murray S. Heterogeneous burden of lung disease in smokers with borderline airflow obstruction. Respir Res. (2018) 19:223. 10.1186/s12931-018-0911-z 30454050 PMC6245799

[32] Maggini S Wintergerst ES Beveridge S Hornig DH. Selected vitamins and trace elements support immune function by strengthening epithelial barriers and cellular and humoral immune responses. Br J Nutr. (2007) 98(Suppl. 1):S29–35. 10.1017/S0007114507832971 17922955

[33] Sezikli M Cetinkaya ZA Guzelbulut F Yesil A Cosgun S Kurdas OO. Supplementing vitamins C and E to standard triple therapy for the eradication of Helicobacter pylori. J Clin Pharm Ther. (2012) 37:282–5. 10.1111/j.1365-2710.2011.01286.x 21740452

[34] Le Marchand L Yoshizawa CN Kolonel LN Hankin JH Goodman MT. Vegetable consumption and lung cancer risk: a population-based case-control study in Hawaii. J Natl Cancer Inst. (1989) 81:1158–64. 10.1093/jnci/81.15.1158 2545891

[35] Wallgren M. Clinical, endocrinological and spermatological studies after endotoxin injection in the boar. Zentralbl Veterinarmed A. (1989) 36:664–75. 10.1111/j.1439-0442.1989.tb00778.x 2514526

[36] Agudo A Esteve MG Pallares C Martinez-Ballarin I Fabregat X Malats N. Vegetable and fruit intake and the risk of lung cancer in women in Barcelona, Spain. Eur J Cancer. (1997) 33:1256–61. 10.1016/S0959-8049(97)00050-6 9301452

[37] Ziegler RG Mason TJ Stemhagen A Hoover R Schoenberg JB Gridley G. Carotenoid intake, vegetables, and the risk of lung cancer among white men in New Jersey. Am J Epidemiol. (1986) 123:1080–93. 10.1093/oxfordjournals.aje.a114336 3706278

[38] Janssen R Vermeer C. Vitamin K deficit and elastolysis theory in pulmonary elasto-degenerative diseases. Med Hypotheses. (2017) 108:38–41. 10.1016/j.mehy.2017.07.029 29055397

[39] Piscaer I van den Ouweland JMW Vermeersch K Reynaert NL Franssen FME Keene S. Low vitamin K status is associated with increased elastin degradation in chronic obstructive pulmonary disease. J Clin Med. (2019) 8:1116. 10.3390/jcm8081116 31357639 PMC6724066

[40] De Moura FF Moursi M Lubowa A Ha B Boy E Oguntona B. Cassava intake and vitamin A status among women and preschool children in Akwa-Ibom, Nigeria. PLoS ONE. (2015) 10:e0129436. 10.1371/journal.pone.0129436 26083382 PMC4470824

[41] Paiva SA Godoy I Vannucchi H Favaro RM Geraldo RR Campana AO. Assessment of vitamin A status in chronic obstructive pulmonary disease patients and healthy smokers. Am J Clin Nutr. (1996) 64:928–34. 10.1093/ajcn/64.6.928 8942419

[42] Park HJ Byun MK Kim HJ Kim JY Kim YI Yoo KH. Dietary vitamin C intake protects against COPD: the Korea national health and nutrition examination survey in 2012. Int J Chron Obstruct Pulmon Dis. (2016) 11:2721–8. 10.2147/COPD.S119448 27843308 PMC5098518

[43] Morichika D Miyahara N Fujii U Taniguchi A Oda N Senoo S. A retinoid X receptor partial agonist attenuates pulmonary emphysema and airway inflammation. Respir Res. (2019) 20:2. 10.1186/s12931-018-0963-0 30606200 PMC6318915

[44] Caram LM Amaral RA Ferrari R Tanni SE Correa CR Paiva SA. Serum vitamin A and inflammatory markers in individuals with and without chronic obstructive pulmonary disease. Mediat Inflamm. (2015) 2015:862086. 10.1155/2015/862086 26339144 PMC4539170

[45] Centers for Disease Control and Prevention National Center for Health Statistics. About the National Health and Nutrition Examination Survey. Hyattsville, MD: U.S. Department of Health and Human Services, Centers for Disease Control and Prevent, 2007-2016. Available online at: http://www.cdc.gov/nchs/nhanes/about_nhanes.htm

[46] U.S. Department of Agriculture, Agricultural Research Service. 2018 USDA Food and Nutrient Database for Dietary Studies 2015-2016. Food Surveys Research Group Home Page. Available online at: www.ars.usda.gov/nea/bhnrc/fsrg 2018, https://www.ars.usda.gov/ARSUserFiles/80400530/pdf/fndds/2015_2016_FNDDS_Doc.pdf

[47] McGowan SE Holmes AJ Smith J Retinoic acid reverses the airway hyperresponsiveness but not the parenchymal defect that is associated with vitamin A deficiency. Am J Physiol Lung Cell Mol Physiol. (2004) 286:L437–44. 10.1152/ajplung.00158.2003 14711804

[48] Timoneda J Rodriguez-Fernandez L Zaragoza R Marin MP Cabezuelo MT Torres L. Vitamin A deficiency and the lung. Nutrients. (2018) 10:1132. 10.3390/nu10091132 30134568 PMC6164133

[49] Glaab T Vogelmeier C Buhl R. Outcome measures in chronic obstructive pulmonary disease (COPD): strengths and limitations. Respir Res. (2010) 11:79. 10.1186/1465-9921-11-79 20565728 PMC2902430

[50] Tilert T Paulose-Ram R Howard D Butler J Lee S Wang MQ. Prevalence and factors associated with self-reported chronic obstructive pulmonary disease among adults aged 40-79: the national health and nutrition examination survey (NHANES) 2007-2012. EC Pulmonol Respir Med. (2018) 7:650–62. 30294723 PMC6169793

[51] Tilert T Dillon C Paulose-Ram R Hnizdo E Doney B. Estimating the U.S. prevalence of chronic obstructive pulmonary disease using pre- and post-bronchodilator spirometry: the national health and nutrition examination survey (NHANES) 2007-2010. Respir Res. (2013) 14:103. 10.1186/1465-9921-14-103 24107140 PMC3854606

[52] Martinez CH Mannino DM Jaimes FA Curtis JL Han MK Hansel NN. Undiagnosed obstructive lung disease in the united states. associated factors and long-term mortality. Ann Am Thorac Soc. (2015) 12:1788–95. 10.1513/AnnalsATS.201506-388OC 26524488 PMC4722830

[53] Block Dietary Data S Information Management S National Cancer I DIETSYS Version 3.0 User's Guide: Health Habits and History Questionnaire, Diet History and Other Risk Factors: Dietary Analysis System. Bethesda, MD The Institute (1994).

